# Hybrid and Single-Component Flexible Aerogels for Biomedical Applications: A Review

**DOI:** 10.3390/gels10010004

**Published:** 2023-12-21

**Authors:** Mateusz Fijalkowski, Azam Ali, Shafqat Qamer, Radek Coufal, Kinga Adach, Stanislav Petrik

**Affiliations:** 1 Department of Advanced Materials, Institute for Nanomaterials, Advanced Technologies and Innovation (CXI), Technical University of Liberec, 461 17 Liberec, Czech Republic; 2 Department of Material Science, Technical University of Liberec, 461 17 Liberec, Czech Republic; 3 Department of Basic Medical Sciences, College of Medicine, Prince Sattam Bin Abdulaziz University, Alkharj 11942, Saudi Arabia; 4 Department of Science and Research, Faulty of Health Studies, Technical University of Liberec, 461 17 Liberec, Czech Republic

**Keywords:** flexible hybrid aerogels, flexible single-component aerogels, flexible scaffolds, biocompatible aerogels, tissue engineering, sustained drug delivery, mechanical properties

## Abstract

The inherent disadvantages of traditional non-flexible aerogels, such as high fragility and moisture sensitivity, severely restrict their applications. To address these issues and make the aerogels efficient, especially for advanced medical applications, different techniques have been used to incorporate flexibility in aerogel materials. In recent years, a great boom in flexible aerogels has been observed, which has enabled them to be used in high-tech biomedical applications. The current study comprises a comprehensive review of the preparation techniques of pure polymeric-based hybrid and single-component aerogels and their use in biomedical applications. The biomedical applications of these hybrid aerogels will also be reviewed and discussed, where the flexible polymeric components in the aerogels provide the main contribution. The combination of highly controlled porosity, large internal surfaces, flexibility, and the ability to conform into 3D interconnected structures support versatile properties, which are required for numerous potential medical applications such as tissue engineering; drug delivery reservoir systems; biomedical implants like heart stents, pacemakers, and artificial heart valves; disease diagnosis; and the development of antibacterial materials. The present review also explores the different mechanical, chemical, and physical properties in numerical values, which are most wanted for the fabrication of different materials used in the biomedical fields.

## 1. Introduction

**Figure 1:**
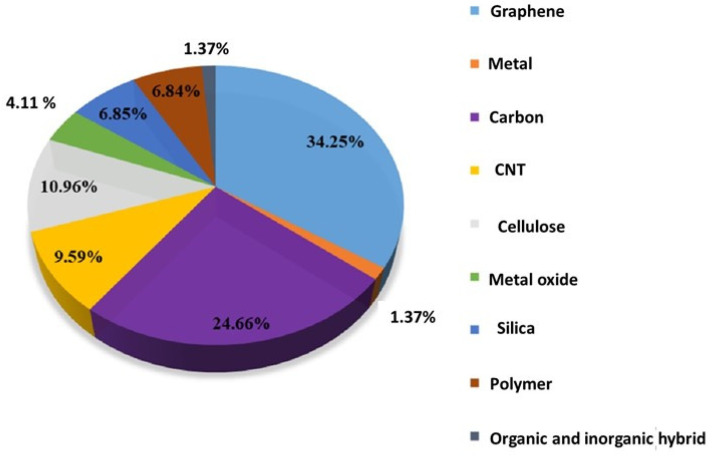
Percentage of aerogels depending on chemical components; data compiled from Web of Science spanning from 2010 to May 2020. Reprint with permission from [Jingyun Jing] (Recent Advances in the Synthesis and Application of Three-Dimensional Graphene-Based Aerogels); published by MDPI (2022) [16].

Aerogels are typically derived from wet gels, and a wide variety of materials, including polymers, biopolymers, and metal oxides, can be turned into aerogels [1]. However, their complicated fabrication, high fragility, and brittle structure greatly limit their practical applications [2]. Hence, the term “flexible aerogels” was introduced [3]. In the case of introducing flexibility by the formation of organic–inorganic structures (such as silica and the organic part of polymers), the prepared aerogels are known as “hybrid flexible aerogels” [4]. Furthermore, some modern research studies are also available concerning the fabrication of hybrid aerogels consisting of two organic polymers. Such aerogels can be called organic flexible hybrid aerogels. The second type, single-component flexible aerogels, is quite a new category composed of single materials that naturally possess mechanical flexibility at a given temperature without the need to be modified by other components. This category consists of pure-form single polymers instead of the commonly used inorganic–inorganic or organic–organic hybrid systems [5]. In the last decade, a large number of aerogels have been produced to meet a wide range of applications. Among them, a major portion has consisted of inorganic-based aerogels (graphene, carbon, carbon nanotubes, etc.). According to a valuation study, the inorganic aerogels have covered about 60% of the application area. Figure 1 describes the estimation of the production of inorganic aerogels. Polymer-based material and biomaterial (e.g., cellulose) aerogels [6,7] are another hot topic, constituting 17.8% of the total literature studies [8]. Among the polymer-based aerogels, the pure polymer aerogels have gained intensive interest in recent years, due to their exceptional flexible properties and attractive applications in the field of energy, sensors, and high-tech biomedical applications [9,10,11].

Significant challenges still persist for the large-scale production, modification, and utilisation of aerogels. While a range of novel synthetic approaches including ambient pressure drying [12] and freeze-drying have been discussed in various research studies [13], pure silica aerogels still have severe mechanical problems and show a lot of limitations in terms of functionality. Thus, various hybrid aerogels, which include organic–inorganic hybrid aerogel systems, have been synthesised in the hope of customising their physical behaviours and functions [14,15]. On the other hand, hybrid flexible aerogels have been produced to meet a wide range of applications. Among them, a major portion has consisted of the hybrid organic–inorganic part, such as carbon, silica, or any inorganic metal in combination with organic polymers. According to an estimate, the hybrid organic–inorganic aerogels have covered about 60% of the application area. 

However, aerogels have boomed in the past decade, and a large number of new aerogels have been designed and synthesised. Figure 1 shows the percentage of the most studied aerogels. Graphene aerogels [17,18,19,20], carbon nanotube (CNT) aerogels [20,21], and carbon aerogels [22,23] are the most investigated topics in the past decade; more than 60% of the literature studies are titled with these carbon materials. Flexible aerogels have shown great potential for multiple biomedical applications. These materials, with their high porosity, large surface area, and low density, offer advantages such as drug delivery, tissue engineering, wound dressing, biosensors, and bio-imaging [24]. They can be used for loading drugs and active biomolecules due to their ability to regulate pore structure [25]. In drug delivery and scaffolds (effective nutrient provider), flexible aerogels play a vital role due to their unique properties such as high stability, porosity, and biocompatibility [26]. Their open structures are most convenient for the sustained release of drugs or broth solution in scaffolds [27]. Additionally, their flexibility and mechanical strength make them the most suited materials to explore for various biomedical applications, including wound healing, tissue engineering, and diagnostics [28]. Biodegradable and biocompatible aerogels, especially those based on polysaccharides, have an advantage over conventional inorganic aerogels [29]. In addition, DNA-based aerogels and DNA-wrapped carbon nanotube composite aerogels have shown potential for applications in real-time computation, neuromorphic computing, biochemical sensing, and biodegradable functional implants [30]. The biomedical applications of aerogels include antifungal, antibacterial, antiviral, biosensing, imaging agents, tissue engineering, diagnosis, and regenerative medicine [31]. Overall, flexible aerogels have the potential to revolutionise various areas of biomedical research and development. 

The present study involves a brief description of the synthesis and properties of hybrid and single-component flexible aerogels. The biomedical applications of these hybrid aerogels are reviewed and discussed, where the flexible polymeric components in the aerogels provide the main contribution. A detailed discussion is made to correlate the novel properties of flexible aerogels (including support to a flexible structure, adaptability, biocompatibility, mobility, comfort for the host (patient), ideal for loading drugs and active biomolecules) with some potential biomedical fields such as tissue regeneration, drug delivery reservoir systems, biomedical implants like pacemakers, artificial heart valves, and stents, heart disease diagnosis, and the development of antimicrobial materials. The investigations and benefits of flexible parts are also explained with systematic schemes and figures. This review will help researchers to further explore the new concepts required for using flexible aerogels in some commodity applications like the development of flexible scaffolds for tissue regeneration. 

## 2. Hybrid Flexible and Single-Component Flexible Aerogels and Their Preparation Methods

The inherent fragility of silica is still a persistent and unavoidable problem in attaining higher mechanical strength in aerogels. Silica-based aerogels inherently exhibit brittleness, which limits their practical applications on a large scale [3]. To address these limitations, the most significant and effective approach is the formation of flexible systems based on organic–inorganic (hybrid flexible), organic–organic (hybrid highly flexible), and pure organic (single flexible) aerogels. In the case of organic–inorganic (hybrid flexible) aerogels, the inorganic component is governed by Si-O-Si interactions, while the organic aspect is characterised by the Si-C bond [3,32]. In the composite system of organic–organic (hybrid highly flexible) aerogels, the aerogels normally involved two different types of polymers and showed significantly different flexibility as compared to the organic–inorganic system; while in the case of pure organic (single-component flexible) aerogels, the synthesised aerogel is composed of only one polymer type. All of these three fabricated systems provide a synergistic effect to enhance the hydrophobicity in aerogels and improve their mechanical robustness [3,33]. Consequently, flexible aerogels are useful in special applications, particularly in medical fields such as medical implantation, tissue engineering, drug delivery, wound healing and tissue regeneration, etc. Presently, flexible and elastic aerogels are synthesised using different approaches such as crosslinking polymers with organosilane compounds, surface modification, and using bridging alkoxysilane precursors [3,32]. The lack of mechanical flexibility in inorganic aerogels has led to the discovery of their organic alternatives. Even though the first organic aerogel was fabricated from cellulose nine decades ago, only a few years back did organic aerogels gain the attention of researchers due to the sustainable nature of the precursors, flexibility, and tuneable surface functionalities [33]. Until now, a wide range of organic flexible aerogels have been made from different precursors, which include biopolymers and synthetic polymers [34]. Alginate, pectin, chitosan, cellulose, and starch are the most important biopolymers used for the fabrication of biopolymer aerogels [33,35].

The stability and performance of flexible aerogel materials depend significantly on the different components that are integrated during the synthesis process [36]. Hence, the preparation of hybrid aerogels primarily follows two main approaches: (i) physical integration and (ii) chemical integration [33]. In the physical integration method, functional components are introduced into the precursor material (whether it be a biopolymer or a synthetic polymer) through mechanisms such as physical entanglements, hydrogen bonding, or van der Waals interactions. While in the case of chemical integration, the functional components are synthesised by using precursor materials, which in turn enhance the interaction between the functional components and the polymer [33].

**Figure 2:**
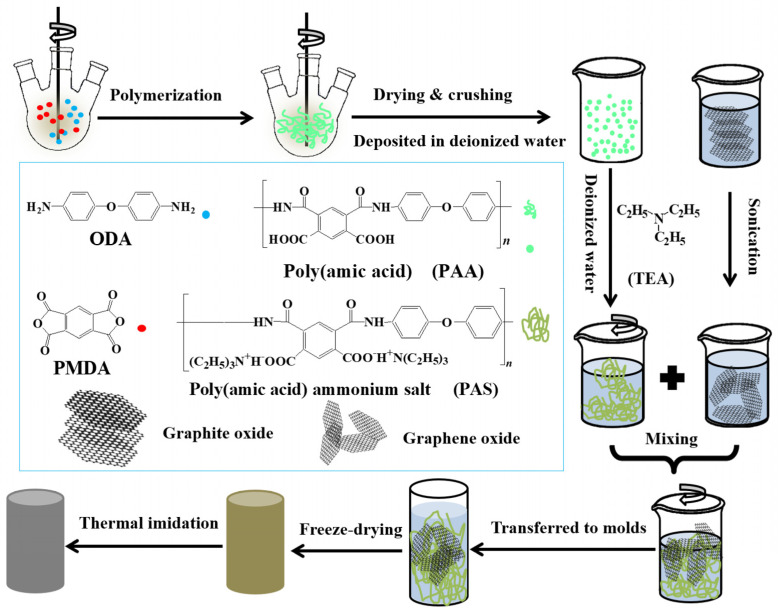
A Schematic diagram illustrating the process for creating GO/PI aerogels. Reproduced with permission from [Li Zhang], [Mechanically Robust and Flexible GO/PI Hybrid Aerogels as Highly Efficient Oil Absorbents]; published by MDPI (2022) [37].

Li Zhang et al. [37] fabricate the GO/PI-based hybrid aerogels through a facile method. Figure 2 shows the preparation methods of GO/PI-based hybrid flexible aerogels. The synthesised flexible aerogels showed higher mechanical properties with a robust flexibility.

**Figure 3:**
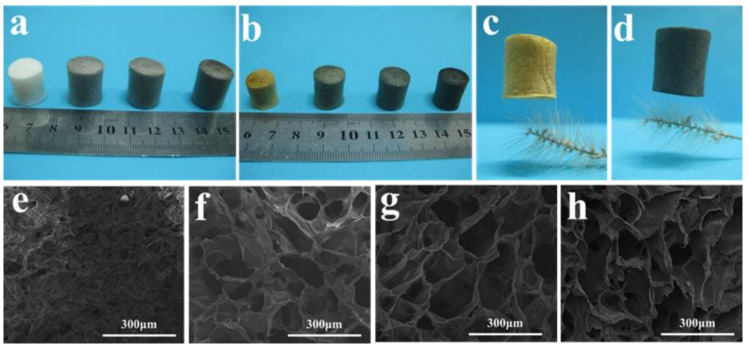
The large-scale and small-scale configurations of GO/PI aerogels. Digital pictures of GO/PI aerogels (**a**) prior to imidization and (**b**) after imidization. Digital images of GIA0 (**c**) and GIA2 (**d**) placed on a bristlegrass. Scanning electron microscope (SEM) images of GIA0 (**e**), GIA0.5 (**f**), GIA1 (**g**), and GIA2 (**h**). Reproduced with permission from [Li Zhang]. [Mechanically Robust and Flexible GO/PI Hybrid Aerogels as Highly Efficient Oil Absorbents] published by MDPI (2022) [37].

During their experimental work, they made a suspension of the water-soluble polyimide (precursor) and graphene oxide (GO) pallets followed by a freeze-drying process, which was further processed through a standard thermal imidization. The resulting porous GO/PI aerogels displayed exceptional elasticity and remarkably low-density values ranging from 33.3 to 38.9 mg·cm^−3^, and they also exhibited a superior compressive strength of 121.7 kPa. The porous structure is obvious from their macroscopic and microscopic digital photographs, as shown in Figure 3a,b. Furthermore, they observed the different concentrations of graphene oxide (GO) in the polyamide polymer. The sample is named GIA0 (meaning pure polyamide i.e., 0 percent of GO). They observed a significant effect of the increase in GO percentage on porosity, as large and looser pores were analysed to be like a honeycomb structure (Figure 3e–h) [37].

**Figure 4:**
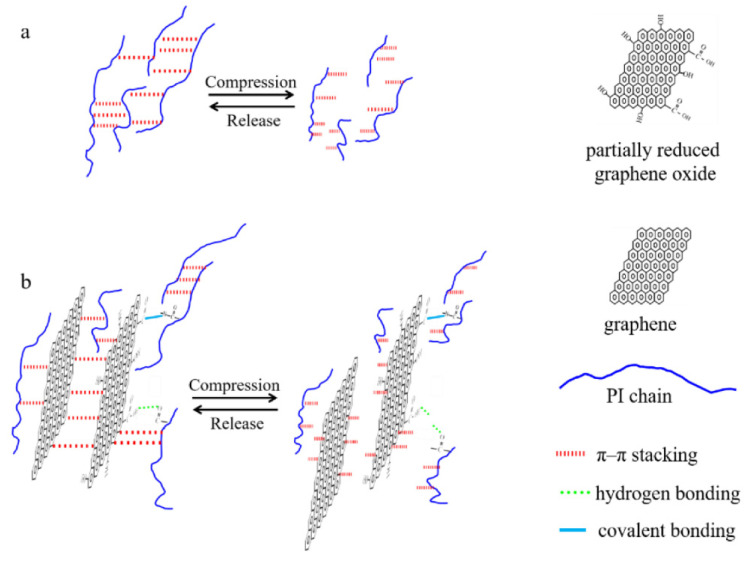
Schematic diagram illustrating the methods for reinforcing and increasing the durability of pure PI aerogel (**a**) and hybrid aerogels (**b**). Reprinted with permission from [Li Zhang], [Mechanically Robust and Flexible GO/PI Hybrid Aerogels as Highly Efficient Oil Absorbents] published by MDPI, 2022 [37].

The resulting aerogels also exhibited an extraordinary weight-carrying capacity, almost capable of supporting a load up to 31,250 times their weight. Their weight-supporting capability was much higher than that of other typical carbon-based aerogels. Their porous structure and remarkable strength and toughness properties make them excellent candidates for materials used in oil spill clean-up. The oil/organic solvents’ absorption capacity ranges from 14.6 to 85, which is higher than that of most other aerogels (sponges). Their ability to tolerate a wide range of temperatures and resistance to highly acidic conditions make these multifunctional GO/PI aerogels highly suitable for application in drug delivery systems and for adverse unhygienic environments [37]. In fact, the high strength and flexibility of fabricated aerogels are due to their unique honeycomb-like microstructures and low densities. These extraordinary properties also showed the synergic effect of covalent and non-covalent bonding between the polyamide chains and the GO nanosheets, as shown in Figure 4a,b [37].

**Figure 5:**
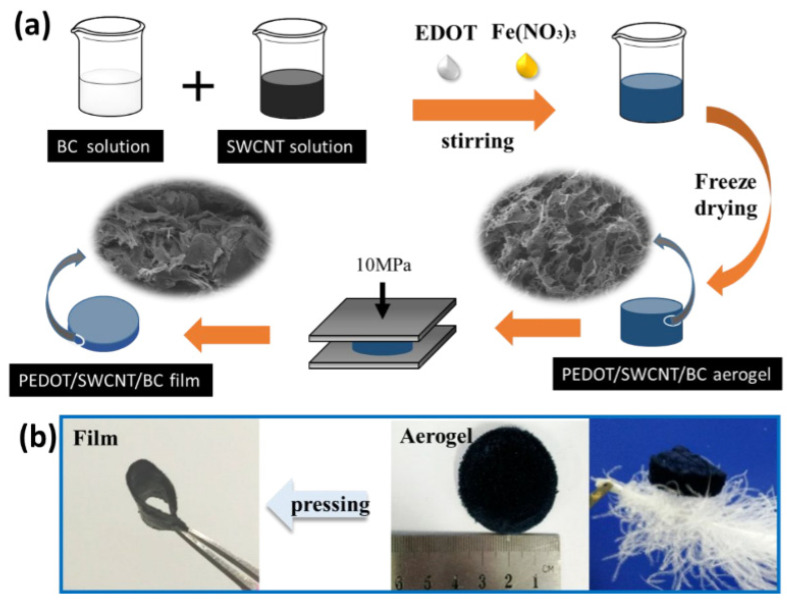
(**a**) Schematics showing the synthesis method for aerogels and flexible, porous PEDOT/SWCNT/BC films from aerogels. (**b**) Digital images of PEDOT/SWCNT/BC aerogel and films, as well as lightweight PEDOT/SWCNT/BC aerogel placed on a fluffy feather. Reprinted with permission from [Fang Jia], [High Thermoelectric and Flexible PEDOT/SWCNT/BC Nanoporous Films Derived from Aerogels]; published by ACS (2019) [38].

Fang et al., [38] created flexible hybrid materials using a chemical process by mixing bacterial cellulose (BC), single-walled carbon nanotubes (SWCNT), iron (Fe^3+^), and a compound called 3,4-ethylenedioxythiophene (EDOT). Afterward, they used a freeze-drying process to make these hybrid aerogels. Subsequently, they fabricated the nano-porous films from these aerogels. The resultant films showed higher electrical conductivity and low thermal conductivity due to the combining PEDOT, SWCNT, and BC in a hybrid mixture. The aerogels and films they prepared were exceptionally flexible, had a fracture strength of 1.6 MPa, and could stretch by 2.13%; the schematic of the materials is shown in Figure 5 [38].

Miao et al. [39] developed an efficient technique to create a special kind of flexible hybrid aerogel. Reduced graphene oxide (rGO) nanosheets and MXene (Ti_3_C_2_T_x_) were used as co-building blocks to create a three-dimensional (3D) flexible hybrid aerogel that was elaborated with ultrafine CuO nanoparticles. The developed 3D MXene/rGO/CuO aerogel is excellent in detecting acetone in the air at room temperature. The resulting behaviour is attributed to the synergistic interactions of highly porous networks and large surface areas. Hence, the copper oxide particles are spread out evenly and provide a high electron conductivity. All these things work together to make it great at sensing acetone. These unique flexible aerogels they created can stretch and bend without breaking [39].

Cellulose nanocrystal (CNC) aerogels are naturally brittle because the crystals are stiff and have restricted mobility. Normally, low-molecular-mass crosslinkers are used to improve the toughness property of aerogels; however, less attention has been paid to making them more flexible. A study by Ling et al. [40] showed a new way to develop nanomaterial-based aerogels with great strength and versatility by mixing functional polymers into the aerogel. They performed the task by using a special type of chemical reaction called click chemistry. In this research, a CNC-PCL hybrid aerogel with enhanced strength and flexibility was synthesised by using a large molecule called polycaprolactone diol (PCL) as a crosslinking agent [40]. Sesan et al. [41] introduced a new way to create a flexible and thermally stable hybrid silica aerogel. The work involved a novel method for fabricating a polyether-based hybrid silica aerogel. Their method was not only fast and easy to scale up but also eliminated the aging process and reduced gelation time.

**Figure 6:**
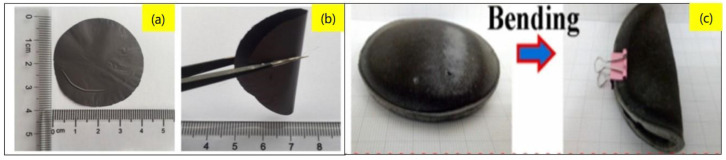
(**a**) Images showing the flexibility of hybrid aerogels. (**b**) twisting, (**c**) bending. Reprinted with permission from [Miao Liu], [Flexible MXene/rGO/CuO hybrid aerogels for high-performance acetone sensing at room temperature]; published by Elsevier (2021) [39,42].

Their novel procedure reduced the solvent exchange and gelation time from many days to just a few seconds. The resulting aerogels showed an outstanding mechanical and flexible performance and excellent thermal insulation properties. Similarly, Xinhai et al. [42] introduced a novel double-crosslinking technique to create a high-strength, freeze-dried polyimide/reduced graphene oxide/cobalt (PI/rGO/Co) aerogel with an interdigitated cellular architecture. The stacking sequence of graphene oxide (GO sheets) (sticks together with hydrogen bonds) and the coordination interactions of the Co ion were responsible for the double-crosslinking. The interdigitated cellular architecture was an important component in producing aerogels with fine densities, improved flexibility, excellent fatigue resistances (1000 cycles), and good mechanical properties (with a compression modulus of 0.506 MPa and a 43% increase in tensile modulus). The developed aerogels were highly flexible in twisting and bending as shown in Figure 6a,b [42].

The flexibility of aerogels is greatly affected by two main factors, i.e., the concentration of hydrogel and the molar ratio of organic polymer to crosslinking agent. According to a study, it was established that the flexibility of the hybrid aerogel is increased when the molar ratio of the crosslinking agent is higher than the organic polymer. It was also concluded that the flexibility of aerogels is also increased by increasing the concentration of hydrogels [43]. Furthermore, some important preparation methods, raw materials, and key properties with areas of application are summarised below in Table 1. 

## 3. Applications of Flexible Aerogels in the Biomedical Fields

**Figure 7:**
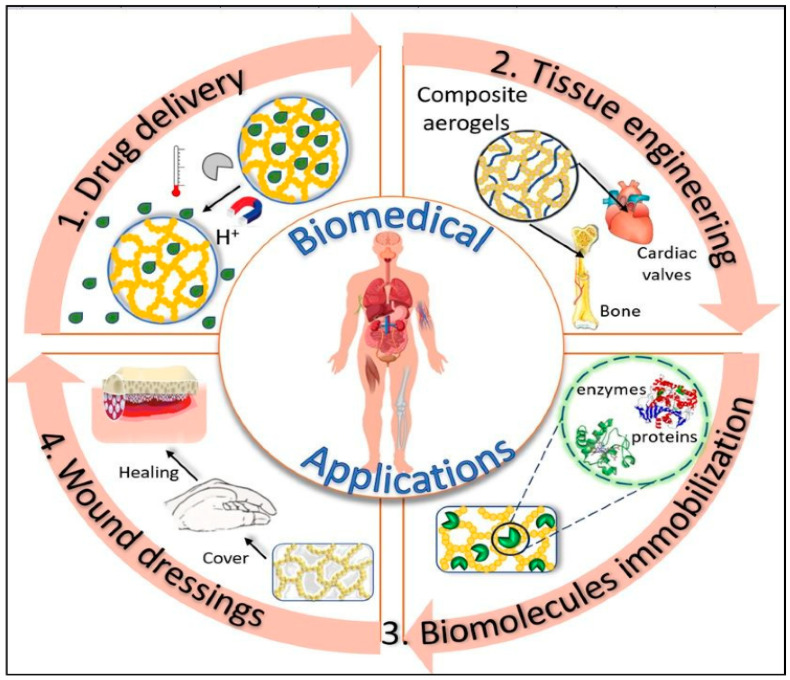
Use of flexible aerogels in different biomedical applications. Reprinted with permission from [Loredana Elena Nita], [New Trends in Bio-Based Aerogels]; published by MDPI (2020) [61].

Over the past ten years, the use of aerogels in the field of biomedical and health sciences has garnered significant attention. The rising demand can be attributed to their unique characteristics such as; highly controlled porosity, large internal surfaces, flexibility and ability to conform to 3D interconnected structures [57,58,59]. Furthermore, biobased aerogels additionally provide exceptional compatibility with living cells, inertness to cells, non-toxic properties, and biocompatibility. These bio-suitable qualities make them excellent candidates for a variety of medical applications such as tissue engineering, drug delivery reservoir systems, biomedical implants like pacemakers, stents, and artificial heart valves, disease diagnosis, and the development of antimicrobial materials [60,61,62,63,64,65,66,67,68,69,70,71,72] (as shown in Figure 7).

The flexible aerogel materials have prompted a growing interest in medical applications for several reasons including providing support to flexible structures [73], adaptability with generating tissues, biocompatibility, mobility and comfort for the host (patient), and being ideal for loading drugs [74] and active biomolecules [75].

## 4. Important Properties of Polymers Used for Biomedical Applications

Different steps and considerations are taken into account during the synthesis of aerogels. The physio-chemical parameters of different polymers play a very significant role in defining the properties of the subsequent aerogel. Some important properties of polymers include the surface area of the polymer [76,77], chemical structure [78], porosity, density, polymer solubility, thermal stability [79], and the hydrophilic or hydrophobic nature of any specific polymer. Generally, for synthesising aerogels, biopolymers are mainly considered to be utilised in biomedical applications. These natural, as well as synthetic, biomedical polymers have been examined thoroughly to test their physical and chemical characteristics in a variety of biomedical applications. Such polymers exhibit tunable characteristics like biodegradation, stimuli responsiveness, and mechanical properties. Various natural polymers such as chitosan, collagen, alginate, cellulose, and gelatin and those from synthetic polymers like poly-lactic acid (PLA), polyethylene glycol (PEG), polycaprolactone (PCL), and poly lactic-co-glycolic acid (PLGA) have been studied for their valuable applications as biosensors, gene delivery vectors, drug delivery systems, and in tissue engineering [80]. The most widely used natural polymers in the field of biomedical applications are bacterial polyesters [81,82] (e.g., bacterial cellulose [83,84]), proteins (e.g., collagen, silk [83,85], gelatin [84], and fibrin), and polysaccharides (e.g., alginate [80], hyaluronic acid [81], and chitosan [82]), which stand out as extensively employed natural polymers in the realm of biomedical applications [86]. These polymers find utility in diverse formats, such as 3D porous scaffolds, hydrogels, nanoparticles, composites, and absorbent sponges [87]. Researchers have delved into modifying existing natural biopolymers and synthesising novel ones to enhance characteristics like solubility, degradation rate, toxicity, immunogenicity, and stability. The current focus of research revolves around synthesising and utilising biomedical polymers, with emerging bioactive polymers, including peptide/bio-membrane/microorganism/cell-based biomedical polymers, showing the potential for precision cancer therapy. Instead of using generic materials, certain biomaterials require specific qualities in polymers. For example, when creating scaffolds, it is essential to incorporate suitable biophysical and structural features like stiffness, roughness, topography, and alignment. Additionally, the inclusion of biochemical signals such as signalling molecules, growth factors, and proteins is crucial. This ensures the promotion of the natural ability of cells to stick, move, multiply, and transform, fostering the development of new tissue. These materials preserve the necessary biochemical cues and properties, enhancing their compatibility with living systems and mimicking the structures found in the extracellular matrix (ECM) of native tissues [88].

As a result, these materials typically exhibit favourable cellular attachment, enhance cellular behaviour, and prevent immunological reactions. However, in certain instances, these properties may be constrained due to variations in batches during production and purification processes.

Nevertheless, the limited mechanical strength of natural polymers often poses challenges during manipulation and the fabrication process. Consequently, it is common to address this issue by incorporating derivatives or blends with different polymers to achieve the necessary mechanical properties for practical use. An illustration of this approach is the modification of gelatin with methacrylamide, resulting in a photopolymerisable biomaterial suitable for applications like 3D bioprinting and microfluidics [89]. Furthermore, the inclusion of various growth factors or other biomolecules has the potential to enhance cell migration, growth, and differentiation [90].

### 4.1. Flexible Aerogels for Wound-Healing Applications

Wound healing is a complex mechanism that involves different cells, cytokines, and genes that contribute to the multistage process of healing [91,92]. The initial inflammatory phase, the proliferative/repair phase, and the remodelling phase are the three stages involved in the healing process for a wound [93]. Some factors such as hypoxia, pH variations, and the growth of pathogens at the injured tissue sites may show a negative impact and hinder the healing process [94]. It is hypothesised that certain bio-supportive conditions may accelerate the wound such as lowering the fluid exudations, promoting the clot-formation factors, and applying antipathogenic treatments at the wound site [95,96,97]. 

Hence, it is assumed that aerogels used for wound dressing would have the ability to absorb exudate and harmful chemicals, allow gas exchange, and inhibit the spread of pathogens. Due to their large surface area, incredibly porous structure, and high permeability, the flexible aerogels can absorb significant amounts of wound exudates, provide control drug delivery, keep the wound site moist, and maintain balance in fluid transportation with control pH in the wound site. Some renowned organic materials including nanocellulose, chitin/chitosan, alginates, and especially collagen have been used for hybrid and single-component flexible aerogels [98,99,100,101]. For instance, Nordli et al. [102] used the combination of sodium hydroxide and TEMPO-mediated oxidation to synthesised ultrapure CNF-based aerogels. The CNF aerogel has a low endotoxin level (45 EU/g) and good endotoxin resistance and can absorb water to a degree that is 4–5 times more than that of alginate-based wound dressings [103]. A study proved the ideal compatibility of CNF-based aerogels with human epidermal keratinocytes and fibroblasts. Their claim was based on deep investigations and comparative analysis with commercial wound dressings like AquaCel^®^. These studies demonstrate that collagen is the main structural protein of the ECM in connective tissues. The function of collagen is to serve as the extracellular matrix (ECM) and promote the process of wound healing. Different collagen-based biomaterials and their composites work as crosslinkers and functional additives to promote tissue regeneration and wound healing [104].

Dharunya et al. [105] reported collagen-based aerogels that were crosslinked with curcumin. The fabricated aerogels showed excellent physical and mechanical properties with enhanced anti-proteolytic and proangiogenic activity. The three-dimensional microstructure of the collagen aerogel was fully crosslinked with curcumin and did not affect structure even by the addition of curcumin. In addition to increasing permeability and water retention, these porous structures also promoted cell adhesion and proliferation. Because of their pro-angiogenic activity and regulated anti-proteolytic activity, the collagen–curcumin aerogels can be used in a diverse range of biomedical areas, including the restoration of injured tissues.

**Figure 8:**
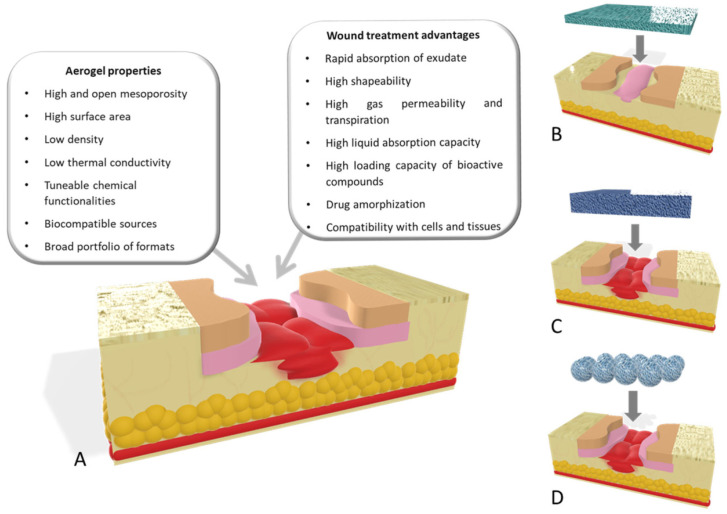
(**A**) Important aerogel properties for wound healing. Different aerogel configurations used: (**B**) aerogel films for wound-healing purposes used in low-thickness wounds; (**C**) scaffolds; and (**D**) aerogel particles as wound agents for managing healing processes for deep wounds. Reprinted with permission from [Beatriz G. Bernardes], [Bioaerogels: Promising Nanostructured Materials in Fluid Management, Healing and Regeneration of Wounds]; published by MDPI (2021) [107].

Govindarajan et al. [106] reported the modified wheatgrass bioactive compounds reinforced collagen-based aerogel. These aerogels worked as an extracellular matrix for collagen turnover and angiogenesis for applications in wound healing [106,107]. The 3D porous architecture of collagen aerogels was further improved with bioactive components from wheatgrass, which in turn increased the physical, chemical, and biomechanical properties of the structure. The 3D aerogel-based proangiogenic and antibacterial bioactive chemical sustained delivery to the cellular microenvironment promotes wound healing. Chitosan, a naturally occurring linear polysaccharide, may be found in the shells of shrimp and other crustaceans. It is a prolific, widely available, and moderately priced material. Chitosan-derived biomaterials have shown promising applications in bioactive materials and wound-healing applications. Figure 8 shows different aerogel properties that are of the utmost importance for wound healing and their effect on the healing process at different stages of healing [108].

Batista et al. [108] created alginate–chitosan aerogel fibres with a highly porous cotton-like structure. They tried to use the components based on biomaterials that are nontoxic and biocompatible. When compared to commercial medical devices (Kaltostats), which were ideal prospects for wound healing, the aerogel fibres had a high antibacterial activity, stimulated cell migration, and exhibited comparable efficacy in cell experiments. In fact, the addition of polymers into the hierarchical framework also results in an excellent performance for biological applications.

Cai et al. [59] used an electro-assembly approach to develop and produce chitin nanoparticle-based freestanding hydrogels. Subsequently, these hydrogels were dried to produce highly porous and strong hybrid aerogels for wound-healing applications. The electro-assembly method was an easy, uncomplicated, and controlled method. Normally, this approach depends on the pH of the chitin nanoparticle solution and the degree of deacetylation of the chitin. The electrical assembly process of chitin nanoparticles is reversible due to the physical assembly properties. Using supercritical CO_2_ drying and freeze-drying, chitin-based aerogel and cryogel have been synthesised. Due to its large SSA, interconnected porous structure, and increased hydrophilicity, chitin aerogel has been shown to accelerate wound healing: see Dharunya et al. [105]. Enhancement of biological activity of wheatgrass in collagen aerogels with enhanced physicochemical and biomechanical performance was observed due to intermolecular interactions between wheatgrass and fibres of collagen. DSC demonstrated improved denaturation temperatures. In addition, hybrid aerogels also possess increased water absorption and substance permeability, allowing channels for various cell growth agents to develop. Furthermore, the cumulative effect of wheatgrass growth factors and collagen molecules increased the angiogenic capacity and collagen production of the hybrid aerogel. The performance was demonstrated by in vivo wound-healing tests and the results indicate that the hybrid aerogel is biocompatible, biodegradable non-adhesive, and suitable for applications in wound healing.

### 4.2. Flexible Aerogel for Tissue Engineering

For the past few decades, the main barriers to using transplanted tissues from both humans and animals for tissue-engineering applications have been a limited availability of donors, a poor immune response, and infection [109,110]. Several studies have been conducted to explore novel methods for the regeneration, restoration, or replacement of damaged tissues using materials from nature [110,111]. Just like any biomaterial used as a framework for tissue engineering, aerogels need certain properties. These include suitable microstructures with linked holes of sizes that permit the integration and formation of blood vessels, as well as biocompatibility. The previous aerogels-based scaffold was showing problems in structural support; they were also hard enough to disrupt with surrounding cells. Thus, the flexible aerogels provide a lightweight robust structural framework within scaffolds. The flexibility of a single-component aerogel provides the scaffold matrix, which can be conformable according to the shape of the generated tissue [112,113]. Their bio-adaptability minimises the disruption of other tissues during surgical implantation. Just like any biomaterial used as a framework for tissue engineering, aerogels must possess specific characteristics. These include suitable microstructures with linked holes of suitable sizes to facilitate tissue integration and vascularisation, along with being biocompatible. In addition to a regulated degradation rate, customised mechanical resilience, and the capacity to be shaped in various forms and sizes, the materials should also possess suitable surface chemistry that permits cells to attach, multiply, differentiate, and ultimately generate a new extracellular matrix [114,115,116]. Silva et al. created aerogels derived from chitin [112] as macro-porous scaffolds for tissue-engineering applications. Chitin was altered to produce a bioactive material and the preparation method for aerogel was also enhanced to boost the porosity of the matrices. Additionally, chitosan-based aerogels have been identified and examined for their potential applications in tissue engineering. In another work, a novel strategy to generate hybrid aerogels using algae and lignin was presented by Quraishi et al. and employed. They utilised CO_2_-induced gelation through solvent exchange, followed by supercritical drying [113]. The aerogels were tested in vitro and in vivo, and the results indicated that they were not toxic and had strong cell adhesion, which is encouraging for their prospective application in tissue engineering [113]. By crosslinking a blend of nanocellulose and collagen using a dialdehyde derivative, Lu et al. [114] produced another aerogel. The result was a biocompatible composite with good physical and microstructural characteristics and stability in the physiological environment. Aerogel production has been demonstrated to involve the incorporation of collagen within the matrix created by cellulose dialdehyde fibres.

**Figure 9:**
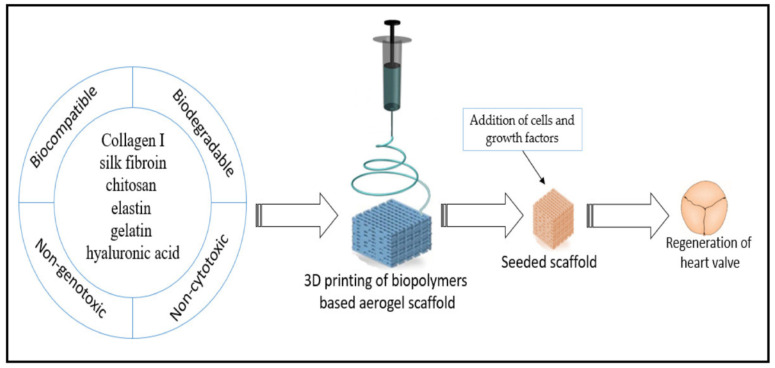
Schematic diagram representing the generation of heart valve, using a biopolymer flexible aerogel scaffold. Reprinted with permission from [Esam Bashir Yahya], [Insights into the Role of Biopolymer Aerogel Scaffolds in Tissue Engineering and Regenerative Medicine]; published by MDPI (2021) [126].

Additionally, the composite flexible aerogel was non-cytotoxic and had remarkable biocompatibility, underscoring the system’s enormous potential use as a scaffold in tissue engineering [115]. A research work was carried out to introduce a material for bone scaffolding and tissue engineering. The scaffold and tissue were constructed using silica gel and poly-ε-caprolactone. Based on the idea that poly-ε-caprolactone (PLC) is a biocompatible polyester that is frequently used in biomedical applications, Ge et al. [116] created a bone replacement that allows the bone cells to promote growth. The effect of silica gel embedding on the final product’s applicability was assessed by the authors. The presence of silica in the porous structure was shown to improve cell survival by preventing any harmful effects caused by the poly-ε-caprolactone (PLC) membrane during prolonged periods of tissue cultivation. Because cardiovascular diseases are the primary global cause of death, intensive research is being conducted on biomaterials with appropriate biological characteristics for cardiovascular implantable devices. However, one major disadvantage of implantable biomaterials is that when they come into contact with blood, they often cause blood clots. This may result in the unintended attachment or deposition of plasma proteins on the material’s surface, which may trigger an acute immunological response. Hence, for use as a cardiovascular implantable device (such as valves), an aerogel must also fulfil several requirements, including low inertia, biocompatibility, and hemocompatibility [117]. These properties are crucial for preserving the valve’s toughness. A macroporous aerogel using polyurea that was nano-encapsulated with silica gels was fabricated by Yin et al. The aerogels were specified for use in cardiovascular applications, and the substances showed high hemocompatibility [118]. These prospective engineering methods for heart valve replacement also showed no alterations in regular platelet activity and showed no acute immune response in blood plasma upon exposure [119]. Many different tissues, including intricate structures like heart valves, may now be grown from scratch using tissue-engineered scaffolds. The engineering of cardiac valve tissues offers a substitute for the traditional, enduring regenerative valve replacement [120]. The main role of the scaffold is to promote tissue regeneration; which supports the tissue formation and remodelling, as well as the slow decay of the biopolymeric film [121]. Wang et al. [122] synthesised the 3D flexible hybrid aerogel-based scaffold, with the combination of collagen and elastin. The authors tried to culture valves by utilising these scaffolds, aiming to create an in vitro three-dimensional valve based on endothelial and interstitial cells [122]. Heart valve regenerations were tried by Fu et al. [123]. They created a scaffold made of chitosan–collagen composites and subsequently seeded it with cells. Staining techniques and histological analysis were employed to distinguish the transplanted cells revealing their identity as smooth muscle cells, fibroblasts, and endothelial cells. According to the authors, these collagen–chitosan composite scaffolds have considerable potential for fostering the growth of heart tissues. This showed that the 6-ketone prostaglandin content was significantly higher in the collagen–chitosan cell culture fluid, as measured through radio-immunoassay. Jahnavi et al. [124] used bovine pericardium, along with polycaprolactone and chitosan. They created bio-hybrid flexible scaffolds to seed a range of human cells and within a few days. Significant extracellular matrix deposition was seen, demonstrating that all of the cells had expanded and multiplied on the scaffold. The authors performed uniaxial mechanical testing on the regenerated valve in the axial direction. They discovered that the scaffolds exhibited a strength that was at least 20 times more powerful and almost 3 times stiffer than native valves. Du et al. [125] achieved similar results in their study; they used a 3D structural scaffold made of silk fibroin and nanofibrous poly (ester urethane) urea for heart valve tissue engineering. The results showed that these fabricated composite scaffolds considerably facilitated the proliferation of human umbilical vein endothelial cells. These biopolymeric scaffolds hold great promise for advancing the field of heart valve development and regeneration. The steps of heart valve regeneration using biopolymer aerogel-based scaffolds are represented in Figure 9.

**Figure 10:**
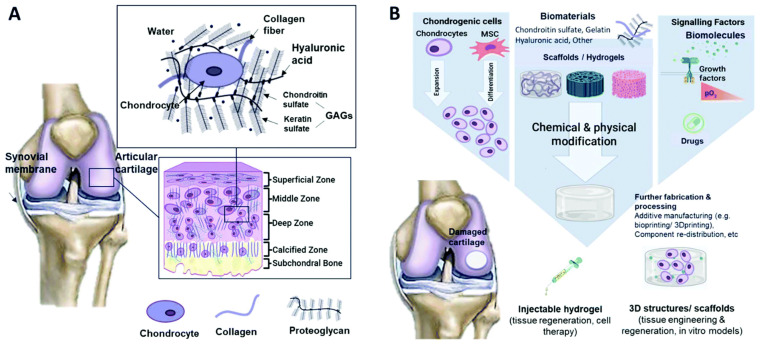
Schematic representation of (**A**) the composition of cartilage and its typical tissue zones and (**B**) the tissue-engineering methodology for cartilage repair. Reprinted with permission from [Mahshid Hafezi], [Biomimetic hydrogels designed for cartilage tissue engineering]; published by MDPI (2021) [128].

Various treatment methods, including surgical procedures such as abrasive chondroplasty, micro-fracture, and cancellation, as well as tissue or cartilage/stem cell grafting, have been used for the reconstruction of articular cartilage in the knee joint (Hunziker et al., 2015) [127]. To grow or regenerate tissues from cells in vitro, tissue-engineering methods can be developed as an alternative to conventional treatments. These methods apply concepts and techniques from multidisciplinary fields such as biological sciences, biotechnology science, chemical engineering, materials science, and mechanical engineering [128]. Cartilage stands out among most tissues because it has no blood vessels, nerves, or lymphatics. Its composition varies across the four regions (Figure 10), which mainly consist of proteoglycans and connective collagen fibres. In the superficial zone, collagen fibres are aligned parallel to the joint surface, whereas in the medial zone, they are not aligned. In the deep zone, the fibres are arranged in a radial direction and finally, in the area of calcified cartilage, a junction is formed between cartilage and bone in the presence of mineralised fibres. The distinct composition and arrangement in each zone layer must be taken into account when replicating natural cartilage tissues, as they contribute unique mechanical properties to each zone [129]. To generate articular cartilage, chondrocytes are seeded onto a biodegradable scaffold of specific shape and volume. The scaffold is escorted by essential growth factors under inducible environmental conditions (Lynn and associates, 2004) [129]. Artificial articular cartilage must assimilate to the existing cartilage and subchondral bone without inducing major inflammatory or immune responses (Kim et al., 2006) [130]. Furthermore, the biomechanical characteristics of artificial cartilage do not match those of the adjacent natural cartilage tissue in terms of its viability and function in the knee joint microenvironment (Malaviya and Nerem, 2002) [131]. Thus, extensive review and a number of preclinical trials are needed before accepting it.

For compounds having a low molecular weight like glucose, oxygen, and growth factors, studies have shown that a pore size of 1 nm is sufficient for their diffusion within polymeric gels that are used in cartilage tissue engineering [132]. However, for compounds having larger molecules such as proteoglycan aggregate, collagen fibre, myoglobin, albumin, and fibrinogen, this pore size is insufficient for free diffusion. Mechanical signals also regulate cell metabolism and induce chondrocytes’ differentiation. Because static loading hinders cartilage synthesis, whereas predetermined cyclic loading enhances ECM synthesis and the suitable development of cartilage, an optimum level of mechanical loading needs to be established to enhance productivity. In addition to mechanical signals, factors such as the characteristics of the hydrogel, cell density, and loading patterns also influence ECM synthesis (Drury and Mooney, 2003) [133].

### 4.3. Role of Flexible Aerogels for Bone Retention

**Figure 11:**
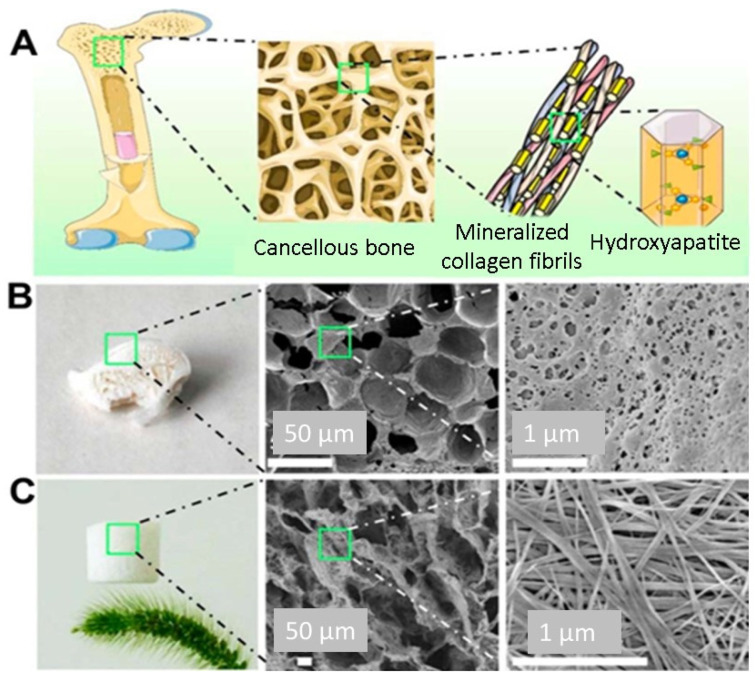
(**A**) Schematic illustration of the microstructure of the cancellous bone. (**B**) Digital image and SEM micrographs of the cancellous bone. (**C**) Digital image and SEM micrographs of the as-prepared HAP nanowire aerogel. Reprinted with permission from [Yong-Gang Zhang], [Bioinspired Ultralight Inorganic Aerogel for Highly Efficient Air Filtration and Oil–Water Separation]; published by ACS (2018) [137].

The bones composite vertebrate skeleton play vital roles in generating red and white blood cells, storing minerals, and providing a structural support system. Bones are robust and long-lasting because of their intricate internal and exterior microstructures. About 70% of the dry weight of the composite is a structure made up of hydroxyapatite (HA) nanocrystals, with the remaining 30% consisting of organic materials including collagen and glycoproteins. Cancellous bones have a porous structure with a porosity ranging from 40% to 90%. They are made up of a continuous web of mineralised organic matrix [134]. Biomaterials based on aerogels feature a 3D interlinked network that resembles the cancellous tissue. Because of their superior biocompatibility and degradability, synthesised HA-based biomaterials are good applicants for bone tissue engineering and repair. Additionally, their bioactivity is similar to that of the inorganic constituents of bones [135]. Recently, a novel solvothermal approach has been used to synthesise hydroxyapatite (HA) tubes containing nanowires and microtubes characterised by significantly high length-to-diameter ratios. These materials have also been used to create a variety of structures, including aerogel structures and ordered structures. Flexible aerogels have also shown promise in the engineering of bone tissue because of their unique characteristics [136]. Flexible aerogels possess the necessary mechanical qualities to resist the needed strength required for applications in bone tissue engineering. In this context, attempts have been undertaken to produce composite aerogels with enhanced mechanical characteristics by reinforcing them with various mixes, like inorganic fillers or biopolymers. Utilising combinations, such lignin, results in improved mechanical characteristics that boost osteoconductivity or cell adhesion. For instance, Zhang et al. employed a novel technique to create a hydroxyapatite (HA) nanowire aerogel with a three-dimensional interconnected and porous meshwork and microstructure to stimulate the microstructure of cancellous bone (Figure 11). The hydroxyapatite (HA) nanowire aerogels displayed high elasticity, low density (8.54 mg/cm^3^) and high porosity (~99.7%). These HA nanowire aerogels are suitable for air filtration and continuous oil–water separation processes. The HA nanowires aerogels showed a porous structure that resembled the mesh-like porosity present in cancellous bones at both the nanoscale and macroscale. As a result, HA nanowire aerogels are considered very promising materials for biomedical uses, especially in the areas of tissue engineering and bone regeneration. In comparison to ordinary HA materials (e.g., HA nanoparticles), HA nanowires have a high aspect ratio, one-dimensional structure, and considerable flexibility. Because of this special quality, it is possible to replicate the natural bone structure by using composite scaffolds in conjunction with materials like collagen and other macromolecules [137].

**Figure 12:**
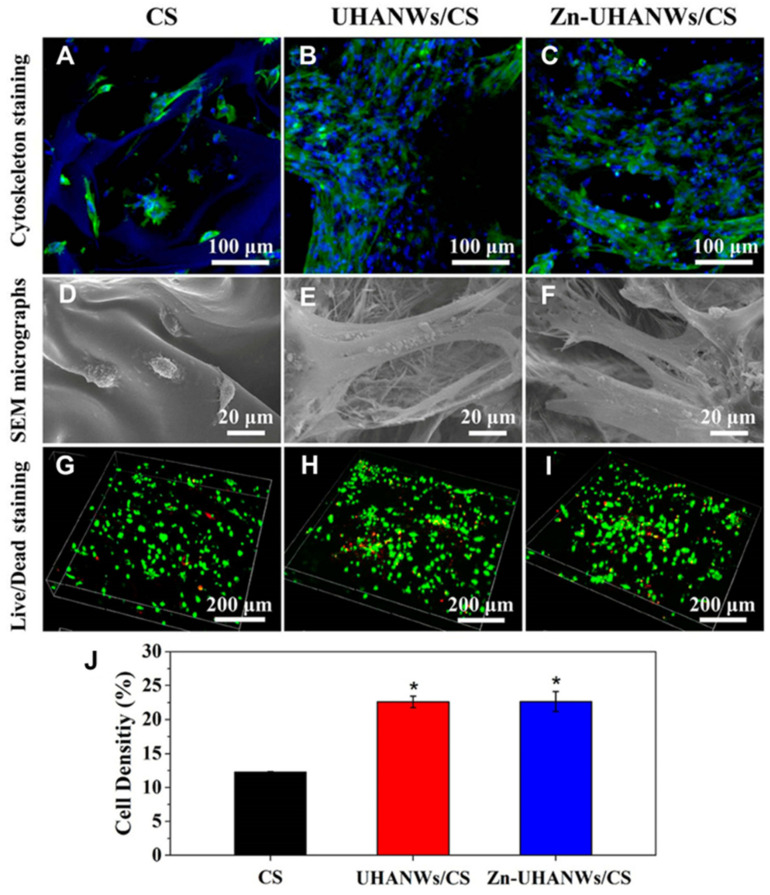
Cell adhesion and growth on the porous scaffolds. (**A**–**C**) Staining of the cytoskeleton and (**D**–**F**) SEM images of rBMSCs on the scaffolds after 3 days of culture: (**A**,**D**) CS, (**B**,**E**) UHANWs/CS, and (**C**,**F**) Zn-UHANWs/CS; (**G**–**I**) staining of dead/alive cells (red staining for dead cells and green staining for live cells) of rBMSCs on the scaffolds after 7 days of culture: (**G**) CS, (**H**) UHANWs/CS, and (**I**) Zn-UHANWs/CS; (**J**) the density of live cells (defined as the green area/total area × 100%) on the porous scaffolds of CS, UHANWs/CS, and Zn-UHANWs/CS. * *p* < 0.05 compared with the CS scaffold group. Reprinted with permission from [Tuan-Wei Sun], [Porous nanocomposite comprising ultralong hydroxyapatite nanowires decorated with zinc containing nanoparticles and chitosan: synthesis and application in bone defect repair], published by Wiley (2018) [139].

In their study, Sun et al. [138] created a scaffold made of HA nanowires and collagen using chemical crosslinking and freeze-drying methods. The scaffold made of collagen aerogels and HA nanowires displayed good mechanical characteristics and may encourage mesenchymal stem cell adhesion and migration. Additionally, experiments conducted in vivo displayed that the scaffold significantly improved bone regeneration. A one-step solvothermal technique was used by Sun et al. [139] to generate zinc-containing HA nanowires. The resulting nanowires have a hierarchically rough surface, as shown in Figure 12, and they may be easily integrated using a freeze-drying technique into composite porous scaffolds with chitosan. The resultant scaffolds stimulated bone regeneration and the differentiation of mesenchymal stem cells produced from rat bone marrow, and they also had improved mechanical qualities. Additionally, Zhang et al. [140] produced ultralight ceramic aerogels made of HA microtubes with an ordered structure. These microtubes had a single HA phase and a uniform size distribution.

**Figure 13:**
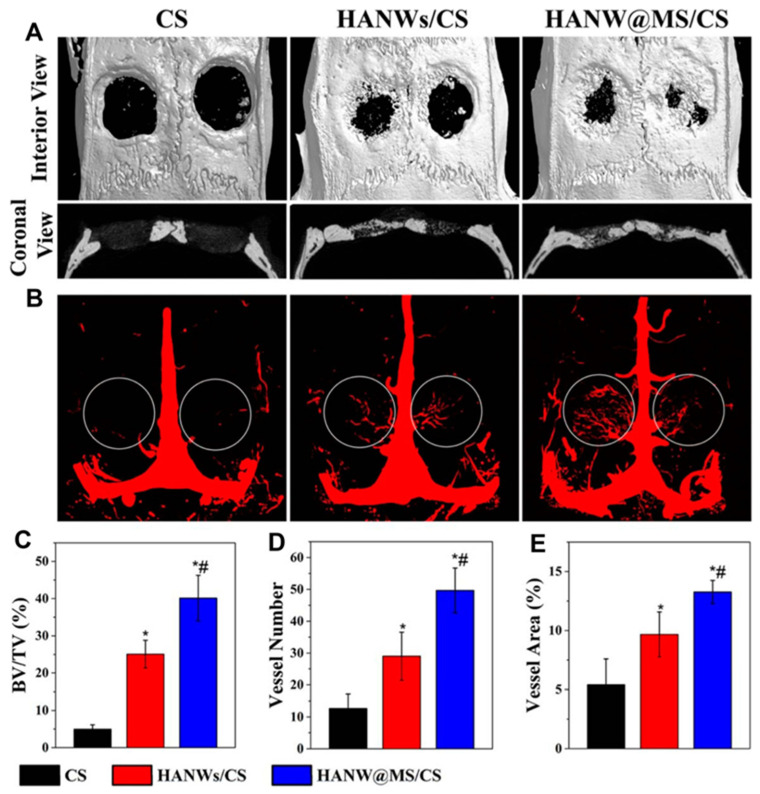
Micro-CT assessment of newly formed bone and blood vessels in rat calvarial defect regions after the implantation of scaffolds of CS, HANWs/CS, and HANW@MS/ CS for 12 weeks. (**A**) Three-dimensional (3D) and coronal views of reconstructed calvarial images. (**B**) Newly formed blood vessels presented by 3D reconstructed images. Morphometric analysis was completed for the percentage of newly formed bone volume (BV/TV) (**C**), blood vessel number (**D**), and blood vessel area (**E**) in the defects. * *p* < 0.05 compared to CS scaffold; # *p* < 0.05 compared to HANWs/CS scaffold. Reprinted with permission from [Tuan-Wei Sun], [Hydroxyapatite nanowire@magnesium silicate core-shell hierarchical nanocomposite: synthesis and application in bone regeneration]; published by ACS (2017) [142].

By using a one-step solvothermal process, it was possible to successfully synthesise a product with high biocompatibility. After the microtubes were subjected to a freeze-drying process and sintered at 1300 °C in an air atmosphere, ceramic aerogels with porous and well-organised structures were achieved. After the sintering process, the distinctive tubular structure remained intact in the resulting HA microtube ceramic aerogels. The HA micro-tube aerogels demonstrated exceptional qualities including high porosities (89–96%), high compressive strengths (up to 1 MPa), ultralow densities (94.1 to 349.9 mg/cm^3^), interconnected tubular channels, an excellent biocompatibility, and high permeability. Additionally, when combined with chitosan through a freeze-drying process, the monodispersed HA microtubes effectively enhanced the mechanical properties of the resulting porous composite scaffolds in comparison to pure chitosan scaffolds and chitosan–HA nanorod composite scaffolds [141]. This was due, in part, to the hollow structure of the HA microtubes, which facilitated drug loading. The composite scaffold made from HA microtubes and chitosan exhibited robust drug release and antibacterial properties over time. The ultralong microtubes composed of HA demonstrated new and promising biomedical applications. In comparison to other reported HA materials, the ceramic aerogels and composite porous scaffolds made from HA microtubes displayed unique physical, chemical, and biological attributes, making them promising contenders for bone regenerative medicine. Biomaterials that possess excellent biocompatibility, bioactivity, biodegradability, and drug-transporting capabilities have great potential as bone-healing solutions. The HA nanowires can also be further enhanced for additional functionality. For instance, by utilising a straightforward template method, the core-shell porous hierarchical HA nanowire@magnesium silicate nanosheets (HANW@MS) nanowires have been created [142]. The core-shell porous hierarchical nanostructures showed noticeably enhanced specific surface areas and pore volumes as compared to HA nanowires, making them ideal for drug loading and sustained release. Therefore, they were deemed suitable for drug loading and extended release. To create the porous scaffold, HANW@MS was combined with chitosan (CS). The resulting HANW@MS/CS scaffolds encouraged stem cell differentiation into osteogenic and angiogenic tissues, as well as cell attachment and proliferation. The HANW@MS/CS porous scaffolds also demonstrated a high bioactive performance, effectively promoting in vivo bone regeneration (Figure 13). The self-assembling HA materials have also been created into porous scaffolds based on aerogel in addition to the one-dimensional structure. Using a microwave-assisted hydrothermal process, copper (Cu)-doped mesoporous HA microspheres (CuMHMs) were created, and they showed a hierarchical structure and a highly focused surface area. The Cu-MHMs were then added to a CS macromolecule scaffold that maintained a high porosity and could constantly release Cu ions. It is significant to note that the in vivo experiment revealed that the Cu-MHM/CS porous scaffolds promoted bone regeneration by concurrently promoting osteogenesis and angiogenesis of the wounded tissue, which was advantageous for the restoration of vascularised tissue-engineered bone. 

The constituent compositions and architectures of natural bones are therefore closely resembled by the as-prepared 3D scaffolds. The outcomes of in vivo implantation further showed that the microstructural orientation of this 3D scaffold considerably supports the repair of bone defects.

### 4.4. Flexible Aerogels for Drug Delivery

The flexible aerogels have gained significant interest in pharmaceutical field, mainly in drug delivery applications. Their physical properties such as flexibility, pores and pore size distribution, open porous structures, and large surface area are all suitable for low-solubility drugs, improved stability, and release kinetics [143]. These properties can be finely adjusted and controlled by modifying the synthesis conditions, making the nanostructured aerogels a promising class of materials for the delivery of a wide range of drugs, as well as enzymes and specific proteins, as has been presented in the specific literature and specialty reviews [144,145,146,147].

The flexibility and ability to customise according to the specific drug delivery needs make the aerogels a valuable material in sustained drug delivery systems. The polymeric-based flexible aerogels performed well as carriers and release systems for a variety of medications (i.e., pharmaceuticals that are difficult to transport via conventional release systems). The high porosity and flexibility of the structure can offer a controlled release of medications on specific sites over a prolonged period [25]. Indeed, the stability of their structures and bioavailability make them suitable for sustained drug delivery. Some single-component (single polymer-based) protein and polysaccharide aerogels obtained from natural sources, such as silk, gelatin, and alginate, have a highly porous network, which enables simple drug release. Their flexibility and shape adaptability allows them to be loaded with drugs through physical entrapment and hydrogen bonding, ensuring precise and controlled release over a long period. 

**Figure 14:**
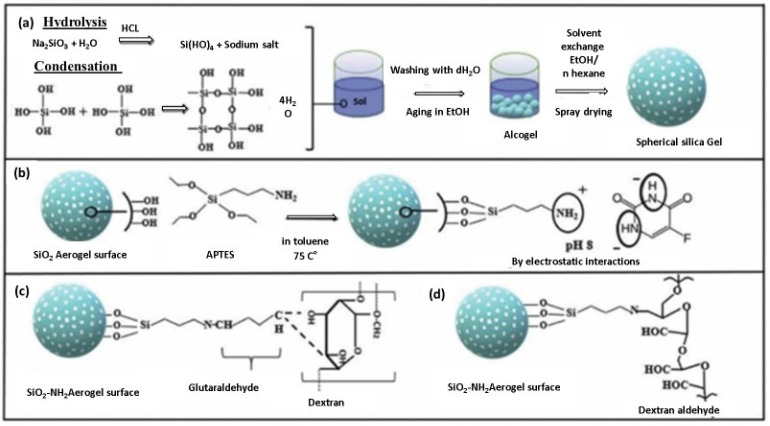
Visual depiction of the experimental procedure: (**a**) Creating spherical silica aerogels through the sol-gel technique, (**b**) modifying the silica aerogel surface with APTES and incorporating the 5-FU drug, (**c**) applying a dextran layer to the silica aerogel surface with GA crosslinker, and (**d**) covering the silica aerogel surface with dextran aldehyde. Reprinted with permission from [Ecem Tiryaki], [Novel organic/inorganic hybrid nanoparticles as enzyme-triggered drug delivery systems: Dextran and Dextran aldehyde coated silica aerogels]; published by Elsevier (2020) [151].

Camptothecin (CPT) is an anticancer medication with superhydrophobic properties that exhibits antitumor efficacy. However, its practical applicability is limited due to its low water solubility [148]. CPT can be delivered and loaded using PVA-PAA hybrid flexible aerogels and methyl-group-functionalised KCC^−1^ nanofibrous silica microparticles. The flexible aerogels sustainably release CPT beyond 14 days. The drug-release profile can be adjusted by adjusting the proportions of KCC^−1^, PVA, and PAA. When applied with various cell lines such as the African green monkey kidney (Vero), immortalised human epithelial (HaCaT), and murine fibroblast (L929), these aerogels decompose with the passage of time. When loaded with CPT, they actively reduce cell proliferation and demonstrate strong anticancer efficacy against a range of cancer cells, including SiHa (HPV16-positive), HeLa (HPV18-positive), and C33A (HPV-negative). The environmentally friendly and biocompatible nature of pH-sensitive hybrid aerogels made from carboxymethyl cellulose (CMC), chitosan, and GO positions make them ideal candidates for drug delivery and loading., Both CMC and chitosan are pH-sensitive polysaccharides that influence the release of 5-FU from CMC/chitosan/Ca_2_+/GO-5-FU. The hybrid aerogels exhibit the highest SR value (20.52) and maximum cumulative release (68%) at pH 7.4, based on controlled 5-FU release and swelling ratios (SR) at different pH levels. The results also emphasise the crucial role of GO in the prolonged release of 5-FU from the hybrid aerogels, with Fickian diffusion governing the release kinetics. This study presents a new opportunity for manufacturing controlled drug carriers using polysaccharides [148]. Mesoporous silica nanoparticle-loaded PVA-PAA hybrid aerogels serve as effective carriers for hydrophobic medications [149]. The hydrophobic anti-inflammatory drug dexamethasone (DEX) serves as a model drug, illustrating the potential of these hybrid aerogels as drug carriers. The aerogels offer a high drug-loading capacity for DEX due to their hydrophobic pores, with an encapsulation efficiency exceeding 75%. By adjusting the PVA-to-PAA weight ratio in the precursors, the release behaviour of DEX can be easily tailored. Additionally, the aerogels provide sustained drug release for at least 50 days [150]. In work by Ecem et al. [151], organic–inorganic hybrid aerogel nanoparticles were designed by coating inorganic silica aerogels with dextran aldehyde (Dex-CHO) and dextran (Dex). These hybrid aerogels enabled the targeted delivery of 5-fluorouracil (5-FU) as enzyme-triggered and colon-targeted materials. Functionalising the silica aerogel surface with 3-(aminopropyl) triethoxysilane (APTES) enhanced the drug-loading and coating efficiency, resulting in minimal drug release (1.7% for Dex-CHO and 3.4% for Dex) compared to uncoated silica aerogels (86.4%). Figure 14 shows the sol-gel process for the synthesis of spherical silica aerogels and their surface modification with APTES. Subsequently, the dextran aldehyde layer to the silica aerogel surface with a GA crosslinker was applied. MTT assay results indicated reduced Caco-2 cell viability but no significant cytotoxic effect. Dex-CHO and Dex-coated silica aerogels prove to be biocompatible nanoparticles ideal for enzyme-triggered drug delivery to the colon region. A multi-layer composite (MLC) combining a drug-loaded silica aerogel powder and a PVA film substrate was developed for fluconazole drug delivery. The hybrid aerogels released fluconazole more rapidly than pure fluconazole, likely due to their high surface area (4800 m^2^ g^−1^) and porosity (480%). PVA nano-fibres regulated the fluconazole release rate, with non-Fickian diffusion governing the drug release mechanism [152]. Coated and uncoated silica-alginate hybrid aerogel beads were created using an emulsification/internal setting technique. These hybrid aerogels combine the high surface area, drug delivery, and mechanical strength of silica with the bioadhesive and biodegradable properties of alginate. The hybrid aerogel beads had a surface area of 900 m^2^ g^−1^ and a particle size of 282 µm. The hydrophobic silica coating or silica-hydroxypropyl methylcellulose affected the loading and release rate of the model drug ketoprofen. The coated hybrid aerogel beads exhibited an extended release lasting up to 60 min. It is worth noting that aerogels can be fabricated as 2D films, 3D bulk monoliths, 1D fibres, and 0D particles [152].

Aerogel particles are a more suitable option for drug delivery. In fact, the micro-sized aerogel particles effectively release as compared to macro-sized monolithic structures. Additionally, these micro-sized or nano-sized aerogels can circulate within the body, which is imperative for controlled and targeted delivery. When considering the applications of aerogels for wound healing and tissue regeneration, the micro or nanosized flexible aerogel structures always exhibit an excellent performance compared to larger bulk aerogels. Additionally, the sizes and structural designs of the aerogel matrix are selected and aligned according to the shape of the specific tissue which they are intended to support [152].

### 4.5. Antibacterial Effect of Flexible Aerogels

In recent research, it has been found that many materials with antibacterial properties have been incorporated into the nanocellulose matrix systems during the fabrication of aerogels. These matrix systems include plant extracts, essential oils, silver nanoparticles, and enzymes, which have been shown to retain their antibacterial activity after being immobilised within the aerogel. Yahya et al. [153] found that the antibacterial activity of Punica granatum peel extract showed a stronger effect of antibacterial activity than commonly used antibiotics. In a study by Khan et al. [154], silver nanoparticles and enzymes were immobilised inside CNF aerogels and evaluated for potential clinical trial applications. They proved that the fabricated aerogels were non-toxic and biodegradable. Dialdehyde CNF/collagen composite aerogels, prepared by Lu et al. [155], showed greater water absorption and biocompatibility. In addition to different functional properties, their antibacterial effectiveness was enhanced with the addition of plant-based antibacterial materials, thus preventing bacterial growth. Shan et al. [156] encapsulated amoxicillin antibiotics within cellulose aerogels and reported excellent antibacterial properties with amoxicillin dose-dependent activity [156]. The encapsulation of antibiotics inside the aerogel network enhances their activity. Cellulose has a unique surface chemistry that allows it to crosslink with many materials. Table 2 describes the summary of applications of different hybrid and single-component flexible aerogels [157].

Zuguang et al. [164] manufactured hybrid cellulose-based aerogels by incorporating silver nanoparticles as antibacterial agents. These hybrid aerogels exhibited potent antibacterial properties against a variety of bacterial strains. To enable strong crosslinking of silver nanoparticles, the authors partially oxidised certain groups and damaged the cellulose crystalline area. This kind of surface modification was applied to cellulose to improve its functionality and stop the potential production of nanoparticles that could have long-term negative effects on human health. Wet-stabilised aerogels that retained their porosity after being soaked in water were developed by Henschen et al. [165]. These cellulose surface-modified aerogels were capable of adhering to more than 99% of bacteria in the aqueous suspension thanks to the surface modifications made to cellulose to allow for bacterial adherence [165].

## 5. Future Perspective

Extensive research has been conducted on the use of porous and incredibly flexible aerogel materials in novel medical applications. The more advanced flexible aerogel types and their preparation methods have been demonstrated to be among the most effective flexible porous materials. More work must be conducted to increase the porosity and flexibility of hydrogels and aerogels. Most aerogel research focuses on preparation techniques and function analysis, which may involve managing structures or properties. Consequently, a great deal of study remains to be carried out on applying aerogels in tissue engineering and wound healing for various uses [166]. Self-repairing aerogels must overcome various difficulties in the future, mainly to advance biomedical applications. These parameters include acceptable mechanical characteristics, clinical trial compliance, and biocompatibility. Moreover, there is a need to optimise the biodegradability of self-healing aerogels that are mostly used in artificial skin, biochemical engineering, and drug-carrying applications. The novel category that is a single component enables the production of highly flexible scaffolds that would not be achievable using traditional aerogel synthesis methods. Comprehensive knowledge about the underlying medical conditions is necessary for the most successful treatment approach. Similarly, synthetic 3D lung tissues formed by the use of graphene-aerogel would allow a fundamental investigation for the realistic modelling of the required systems [167]. To solve some limitations of the 3D-printing technology, it is necessary to investigate the complex dynamics of natural tissue products and also the optimisation of cell-construct functional responses [168]. With the beginning of bioengineering technology, the production of biopolymer-based scaffolds by using aerogel-based material and 4D-printing technology is interesting to discover. Hence, such biopolymers may become significant components in formulations of bio-ink in the future. This is because they highly relate to 4D bio-printing for the purpose of gene therapy and the bioengineering of tissue [169]. Despite some aerogels being considered safe because of their ingredients, reports of harmful incidents have rarely emerged. To avoid toxicity, the original aerogel has occasionally needed to be altered with more biocompatible components [170]. Consequently, highly flexible, highly porous aerogels without standing mechanical properties should be synthesised for hi-tech biomedical applications. That not only excludes any possible hazardous occurrence but also provides flexible complex drug delivery tools, implants, and scaffolds with enhanced structural design and boosted biocompatibility [171].

## 6. Conclusions

This review reports the state-of-the-art fabrication of hybrid and single-component flexible aerogels and their potential applications in the medical area. Over the past decade, the interest in using flexible aerogels in medical fields is increasing rapidly; more than 70% of the references cited in the present study are post-2013. The review starts with a concise summary of the synthesis of organic–inorganic, organic–organic, and pure organic (single component) flexible aerogels; followed by describing the benefits of their flexible parts. The special class of flexible aerogels (single-component flexible aerogels) showed the tendency for higher mechanical properties along with excellent flexibility and high porosity. Moreover, it was analysed that the flexible structured features of aerogels such as their highly controlled porosity, large internal surfaces, flexibility, and ability to conform into 3D interconnected structures make them excellent candidates for a variety of medical applications.

Moving forward, a detailed discussion was made to correlate the novel properties of flexible aerogels (including support to a flexible structure, adaptability, biocompatibility, mobility, comfort for the host (patient), and being ideal for loading drugs and active biomolecules) with some potential biomedical fields such as tissue engineering, drug delivery reservoir systems, biomedical implants like pacemakers, stents, and artificial heart valves, disease diagnosis, and the development of antibacterial materials. The investigations and benefits of flexible parts were well explained with systematic schemes. This review will help researchers to further explore the new concepts required for using flexible aerogels in some commodity applications like the development of flexible scaffolds for tissue regeneration, as a range of tissue regenerations such as bone, skin, heart valves, cartilage, and heart valves have been the main challenges associated with biomedical engineering. After an intensive review, it is also concluded that there have been few studies investigating the effects of flexible aerogels. Thus, in the future, there is still a broad gap to improve the specific targeted properties of flexible aerogels and make them suitable for further commodity medical applications.

## Figures and Tables

**Table 1:** Categories with preparation methods, raw materials, and key properties of some synthesised aerogels.

Category of Aerogel	Raw Materials	Preparation Methods	Important Key Properties	Area of Applications	Ref.
Single component	polyimide-based aerogels	Chemical synthesis with Desmodur N3300A crosslinkers	Higher tensile strength and compression properties	High-strength porous polymeric applications	[44]
Single component	polyimide-based aerogels	Crosslinkers having a backbone of diamine	Highly flexible	Highly flexible and high-strength applications	[45]
Single component	polyolefin-based	Solvent evaporation	High compressive strength	Mechanically stable grained structures	[46]
Hybrid Organic–organic	DMDMS with MTMS	Sol-gel process in Two-step and SCD	Aerogels with high degree of flexibility showing spring back behavior stress (~0.10 MPa)	Flexible electrical devices Protective gear	[47]
Hybrid Organic–organic	TEOS and aramid fibres	Sol-gel method in one-step and APD	Thermal conductivity of flexible aerogels ranging from 0.0221 to 0.0235 W/m·K	Pipe cooling Building insulation	[48]
Hybrid Organic–inorganic	Triisocyanate with silane	Drying with supercritical CO_2_	low porosity (76–83%), low bulk density (0.25–0.33 g/cm^3^) and Young’s modulus (30–70 MPa).	Insulations Oil spill clean-up Air filters	[49]
Hybrid Organic–inorganic	MTES Methyl- triethoxysilane	Sol-gel two-step process and SAD	Superhydrophobic, elastic, and flexible aerogels with a Young’s modulus of about 3.95 × 10^4^ N/m^2^.	Impact absorption Structural components	[50]
Hybrid Organic–inorganic	MTES methyltriethoxysilane	Sol-gel process in two-step and SAD	Hydrophobic, light-weight, and flexible silica aerogels Maximal stress ~15.09 kPa	Portable shelters Electronics protection Marine equipment	[51]
Single component	Aromatic diamine	Drying with supercritical CO_2_	Flexible	Flexible applications	[52]
Hybrid Organic–inorganic	VTMS vinyltrimethoxysilane	Sol-gel process in two steps utilising surfactant and SCD (supercritical CO_2_ drying)	Aerogels are transparent and flexible, with 50% compression and nearly 100% durability. Thermal conductivity ~0.0153 W/m·K	Impact absorption Vibration dampening	[53]
Hybrid Organic–organic	TEOS and polyrotaxane	Sol-gel process with one-pot base catalysis and SCD	Transparent and flexible aerogels with density ~0.15–0.30 g/cm^3^ low thermal conductivity 0.012–0.015 W/m·K,	Building insulation Solar energy Medical applications	[54]
Hybrid Organic–organic	MTMS and GPTMS	Sol-gel process in two-step and SCD	100% recoverable flexible aerogels, Young’s modulus of about 0.46 MPa Thermal conductivity ~0.0336 W/m·K	Flexible electrodes Sensors Smart textile	[55]
Single component	MTMS	Sol-gel process in single-step and SCD supercritical CO_2_ drying	80% linear compression and 95% recoverability in transparent and flexible aerogels	Medical devices Pressure relief mattresses	[56]

**Table 2:** Applications of different hybrid and single component flexible aerogels.

Applications	Materials	Summary	Ref
Tissue scaffolds	CNF/PEGDA aerogel	Possessed excellent mechanical and biocompatibility, tested cells tightly adhered and spread on the aerogel, demonstrating excellent differentiation and viability.	[158]
	Cellulose/gelatin aerogel	The surface-altered scaffold for skin regeneration showed good adhesion and proliferation of keratinocytes during the 7-day incubation period.	[159]
	Pure CNC aerogel	Various scaffold structures were successfully printed through the direct ink write technique.	[160]
In drug delivery	CNF aerogels	Used in the realm of controlled drug delivery and resulted in improved drug bioavailability.	[67]
	Cellulose triacetate base aerogel	Tested the influences of the size and the distribution of drugs, aerogels show high drug uptake and gradual release rate	[145]
	Cellulose aerogels	Assessed the loading capacity and release kinetics, promising controlled drug release carriers by exhibiting a high-loading capacity	[146]
Skin tissue repair	Bacterial cellulose	The using of aerogel had a faster and more efficient healing effect and reduced inflammatory response.	[112]
	Nano cellulose aerogel	The novel invention offers a promising process to fabricate bilayer aerogel for skin repair.	[113]
	Amoxicillin/Cellulose aerogels	Grafted amoxicillin onto the cellulose and observed enhancement of the antimicrobial activity against fungus bacteria.	[156,160]
Biosensing	Peptide/cellulosic aerogels	Effectively used a micro-titre enzyme assay to determine the response, sensitivity, and kinetic behaviour of the biosensor.	[161]
	Prussian blue/cellulose aerogel	Tested as an orally administered drug, which was able to detect and remove ingested caesium ions from the gastrointestinal tract.	[162]
	CNC aerogel	Evaluated a sensor to detect human neutrophil elastase for healing chronic wounds.	[163]

## Data Availability

Not applicable.
